# An interpretive phenomenological study: podolinguistics, sportsmen and masculinity

**DOI:** 10.1186/1757-1146-7-S2-A5

**Published:** 2014-11-14

**Authors:** Christopher Morriss-Roberts

**Affiliations:** 1 School of Health Sciences, University of Brighton, Brighton, UK

## Background

This empirical piece of 2013 doctoral research, considers the role of podolinguistics and its relationship to masculinity and sport. Podolinguistics or podopsychosomatics a term originally introduced by Rossi [1] is said to be the language of feet and shoes. Taking this concept a step further, podolinguistics is reading the transmitted signifiers that are understood through wearing different types of footwear, in this instance. In this body of work, the author forms a podolinguistic theoretical relationship to men in sporting environments, with a particular focus on reading into the signifiers of masculinity, utilising an Interpretive Phenomenology and a Masculinity Theory approach.

## Methods

Ethical approval was gained from the University of East London. Eight sportsmen (four heterosexual and four homosexual) were recruited into the study, from a range of different sporting disciplines. Each participant took part in a one-hour semi-structured interview, where insights into masculinity and sport were explored. The structure of these interviews took into account the life histories of the participant. The interviews were transcribed for analysis. An Interpretive Phenomenological Approach [2] was utilised to analyse the data, appreciating the phenomenological, the hermeneutic and idiographic qualities of the interview transcripts, resulting in the development of themes.

## Results

The results indicated a complex relationship of understanding footwear and podolinguistics in the sporting setting. This relationship includes the realisation of three categories of footwear in sport that shape masculine bonds between men. These categories include, sporting-footwear, social-sporting-footwear and general footwear. Developing this categorisation further, it became apparent that colour of sporting-footwear and social-sporting footwear, was the most fundamental/influential feature in shaping/understanding masculinity through a podolinguistic signifying relationship. Finally, the results also indicated that the foot and shoe was not the only feature to be included in the definition of podolinguistics. This empirical piece of research complemented Rossi's [1] definition, by adding the role of 'walking' to the 1977 understanding of podolinguistics. It was found that the role of walking, and modelling a heterosexual stance of walking, was interpreted as masculine/heterosexual.

## Conclusion

This piece of research puts the previously ignored concept of podolinguistics back into the academic frame. In this example, by focussing on masculinity and podolinguistics in sport, we are able to see how a new awareness of this relationship, shapes men’s understandings of their lives and masculinity though footwear. It gives us the foundation in podiatry, to step back and reconsider the psychosocial role of footwear, feet and walking in shaping podolinguistics, as a new academic discipline for debate.
